# The role of stereoradiography in the evaluation of lower limb deformities

**DOI:** 10.1590/0100-3984.2021.0104

**Published:** 2022

**Authors:** Flávio Duarte Silva, Renan Nogueira Chemin, Alípio Gomes Ormond Filho, Júlio Brandão Guimarães, Fernando Ometto Zorzenoni, Marcelo Astolfi Caetano Nico

**Affiliations:** 1 Departamento de Radiologia Musculoesquelética do Grupo Fleury Medicina e Saúde, São Paulo, SP, Brasil.

**Keywords:** Radiography, Lower limb, Lower extremity deformities, Diagnostic imaging, Radiografia, Membro inferior, Deformidades congênitas dos membros, Diagnóstico por imagem

## Abstract

Deformities of the lower limbs are a common condition and can lead to changes in gait, as well as affecting the function and longevity of the hips, knees, and spine. A systematic approach is essential to achieve the desired therapeutic result with the lowest rate of complications. Panoramic radiography is a widely available, low-cost method that is commonly used in order to assess the length and angular deformities of the lower limbs, by measuring the length and angular deviations of the axes. However, because the combination of lower limb deformities in two or three orthogonal planes is common, conventional radiography lacks accuracy because it is a two-dimensional imaging method. Therefore, the measurements of valgus/varus deformities on X-rays restricted to the coronal plane will present increasing variations in measurements depending on the degree of flexion/recurvatum alignment, anomalous bone torsions, or, last but not least, inappropriate patient positioning. Low-dose biplanar stereoradiography using three-dimensional models increases the accuracy of the measurement of several parameters used in the evaluation of lower limb alignment, including lengths, axes, and tibial/femoral torsions, parameters that could previously be evaluated only by computed tomography. Stereoradiography also makes it possible to perform a head-to-toe evaluation, as well as to evaluate the interactions among the lower limbs, pelvis, and spine.

## Abstract (pt)

As deformidades dos membros inferiores são uma condição comum e podem levar a alterações da marcha e afetar a função e longevidade das articulações do quadril, do joelho e da coluna. Uma abordagem diagnóstica sistemática é essencial para definir o tratamento e alcançar o resultado terapêutico desejado com a menor taxa de complicações. A radiografia panorâmica é frequentemente utilizada para caracterizar as deformidades dos membros inferiores por meio de medidas de comprimento e desvios angulares dos eixos, além de se tratar de um método de baixo custo e alta disponibilidade. No entanto, como é frequente a combinação de deformidades em dois ou três planos ortogonais dos membros, a avaliação radiográfica perde acurácia por se tratar de um método de imagem bidimensional. Nesse sentido, deformidades em valgo/varo avaliadas radiograficamente no plano coronal apresentarão variações crescentes nas medidas dependendo do grau de flexão/*recurvatum*, torções ósseas anômalas ou, não menos importante, um posicionamento inadequado. A estereorradiografia biplanar de baixa dose, por meio de modelos tridimensionais, permite obter medidas mais acuradas de vários parâmetros usados na avaliação das deformidades dos membros inferiores, incluindo comprimentos, eixos e as torções tibial e femoral, antes disponíveis apenas pela tomografia computadorizada, com a vantagem de ser realizada em posição funcional com carga. Além disso, por permitir uma avaliação global da cabeça aos pés, abre uma nova perspectiva de compreender a inter-relação das deformidades dos membros com o posicionamento da bacia e com as deformidades da coluna.

## INTRODUCTION

Deformities of the lower limbs can originate from soft-tissue disorders (musculotendinous, capsuloligamentous, chondral, or meniscal disorders) or from the bone tissue itself, and can be developmental or acquired^(1)^. Axis deviations in the coronal plane are commonly observed in developing children and are usually corrected by physiological mechanisms typical of growth^(2)^. However, when such deviations persist, they can lead to gait dysfunction, predisposing to joint changes such as chondropathy, instability, and, occasionally, early osteoarthritis^(3)^. Among the acquired causes of axis deviations in pediatric patients, trauma is the most common; trauma can cause deformities due to fractures, especially physeal or transphyseal fractures, repetitive stress on the growth plate, or osteochondral injuries^(1)^. In adults, the predominant cause of such deviations is degenerative joint deformity (osteoarthritis), which is associated with deviation of the mechanical axes of the lower limbs, creating a vicious cycle that results in progression of the deformity and degeneration^(4,5)^.

Difficulties in establishing an accurate diagnosis and treatment plan often stem from divergences between the physical examination findings and the imaging findings. To understand the origin of divergences and errors arising from the radiographic evaluation, Lazennec et al.^(6)^ conducted a study using measurements of the femorotibial angle, defined as the angle between the mechanical axes of the femur and tibia, in order to characterize valgus and varus deformities of the knees. The authors found a divergence of almost 6° in the coronal mechanical axis measurements in patients who exhibited more than 7° of concurrent flexion or recurvatum alignment. In 12% of the extremities evaluated by the authors, the varus/valgus alignment of the knees in the three-dimensional (3D) images was completely opposite of that observed in the two-dimensional (2D) images, diverging 0.15° in varus/valgus for each degree of increase in femoral torsion and 0.05° for each degree of increase in tibial torsion. In another study designed to show the magnitude of error of coronal angular measurements on radiography^(7)^, with rotations and twists varying from -20° to +20°, recorded a change in the mean values for the lateral distal mechanical femoral angle (from 90.6° to 86.8°), the medial proximal tibial angle (from 90.3° to 88.5°), and the distal lateral mechanical tibial angle (from 98.9° to 90.5°).

In cases in which the deformity is combined in two or three planes (axial, coronal, and sagittal), the difference between the measured and actual severity of the deformity is significant^(8)^, potentially changing the planning of the osteotomy.

## PREVALENCE OF COMBINED DEFORMITIES

The concurrence of changes in more than one plane has been observed in the pediatric population. For example, among adolescent males, the reported incidence of coronal deformity accompanied by physiological flexion is close to 4%^(9)^. Coronal deformity can also occur concomitantly with torsional alterations^(10)^. From adolescence onwards, recurvatum alignment becomes more pronounced and more prevalent, the reported prevalence being 10-12% in young adults^(11)^, and reaching up to 38% in individuals between 40 and 60 years of age^(12)^. Among young adults, the prevalence of alterations in tibial torsion (mostly an increase in external tibial torsion) in conjunction with tibia vara has been shown to be 46%^(7)^.

Another subpopulation to pay special attention to when evaluating the deformity is that of adults and elderly people with severe osteoarthritis, as this condition often involves muscle contractures, which result in some degree of flexion or hyperextension. The prevalence of the loss of full knee extension has been reported to range from 8% in elderly people with good functional status to 75% in the institutionalized population, and such loss is primarily unilateral^(13)^.

## FEATURES OF STEREORADIOGRAPHY

Biplanar stereoradiography is an imaging system performed in a standardized booth, with two detectors arranged orthogonally in an “L” and two collimated X-ray emitters that allow head-to-toe scanning, providing simultaneous frontal and profile images of a patient in the standing position, with a single scan (in 20 s for full body for adults and 15 s for children) that produces a seamless, life-size image (on a 1:1 scale) with no vertical distortion. The system uses a gas chamber interposed between the X-ray emitting tube and the detector (Charpak chamber) which multiplies the quantity of photons that will sensitize the detector. That principle, which earned the Nobel Prize in Physics in 1992^(14)^, is responsible for the lower radiation dose—85% lower than with conventional radiography with the low-dose technique and 96% lower with the micro-dose technique; that is, 2.6 µSv in stereoradiography versus 67.5 µSv in conventional radiography^(15,16)^—as well as for the image quality, which is similar or superior to that of conventional radiography^(15)^.

**Figure 1:**
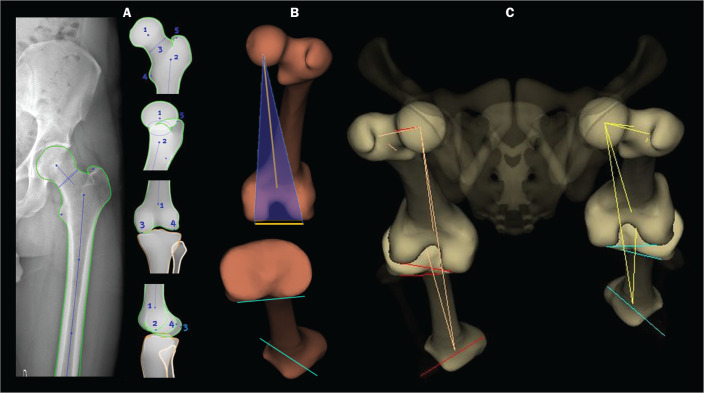
Femoral and tibial reference axes are automatically determined and projected in the axial plane to identify femoral and tibial torsions, respectively, from the 3D models. The reference axes of the femur include a proximal axis, which passes through the center of the femoral head and the center of the base of the neck (A), and a distal axis, which is tangential to the posterior aspect of the femoral condyles (B). The reference axes of the tibia also include a proximal axis, which is tangential to the posterior aspect of the tibial condyles (A), and a distal axis, which is the line joining the center of the malleolus (B). The frontal plane in stereoradiography is defined by the center of the femoral head and the tangent line to the posterior aspect of the femoral condyles (blue triangle in B). C: View of the lower limbs from above.

After some reference points have been demarcated in a 2D image, integrated software uses the simultaneity and orthogonality of the images to generate a 3D model of the bone envelope^(17)^. These 3D anatomy models provide measurements of lengths, angular deviations of the axes, and bone torsion (of the femur or tibia), as well as of the femorotibial rotation, which is the rotation of the tibia in relation to the femur in the knee. The length of the femur is measured from the center of the femoral head to the center of the intercondylar notch, whereas that of the tibia is measured from the center of the tibial plateau to the center of the tibial plafond^(17)^. The sum of the femoral and tibial lengths results in the anatomical length of the limb and does not include the thickness of the knee joint space. The functional length of the limb is the direct measurement from the center of the femoral head to the center of the tibial plafond, including the thickness of the knee joint space^(18)^. On stereoradiography (Figure 1), the frontal plane is defined by the tangent line that passes posteriorly to the femoral condyles^(17)^.

The measurements performed by stereoradiography largely follow those defined in the literature for conventional radiography^(18)^, with some particularities such as the pelvic tilt, which, in stereoradiography, is measured between the acetabula, using a horizontal line tangential to the acetabular roofs as a reference, similar to the line of horizontal femoral reference described by O’Brien et al.^(19)^. Measured in this way, the pelvic tilt indirectly reflects the difference in the functional lengths of the limbs (including the foot height), which is not true for the tilt referenced in the iliac crests^(20)^.

On panoramic radiographs, the assessment of the mechanical axes can be inaccurate (deformities in more than one plane); even on supine radiographs or computed tomography (CT) scanograms, the length can also be measured incorrectly, either by inherent radiographic magnification or by the presence of fixed flexion or recurvatum alignment^(21,22)^.

Stereoradiography is a good alternative to CT when evaluation of limb torsion is required, with the advantage of using less ionizing radiation and the possibility of integrating data on coronal and sagittal axis deviations, because it is performed in a functional position with weight-bearing^(23)^. Panoramic radiography and CT can both allow poor positioning of the feet and lower limbs during image acquisition^(24-26)^, including rotation of the hips, knees, and ankles, which would affect the proper measurement of the femorotibial angle^(17)^. The technical characteristics of biplanar stereoradiography control most of those factors. In a study evaluating 30 femurs in neutral positions^(8)^, with 10° of abduction, 10° of adduction, 5° of flexion, and 10° of flexion, demonstrated the accuracy of stereoradiography for the characterization of femoral torsion as independent of femoral positioning, showing that it is preferable to CT. The authors of that study found that, on CT, the angle of femoral torsion varied with the position of the femur, and hip flexion significantly reduced that angle.

Comparative studies evaluating the torsional profile have shown that stereoradiography was associated with shorter examination times, greater accuracy, and an up to 95% reduction in radiation exposure in comparison with CT^(23,27)^. Another important factor that may result in a lower rate of errors related to stereoradiography is the single exposure for simultaneous acquisition in two orthogonal planes, which reduces the number of movements between one acquisition and another^(17)^. Stereoradiography has some limitations. For example, its image quality for fine bone details is lower than is that of conventional radiography, although that does not change the overall assessment or angular measurements^(28)^. Like conventional radiography and CT, stereoradiography has no sensitivity in soft tissues or ligaments. The measures of patellar translation—the tibial tuberosity-trochlear groove (TT-TG) distance—have not been validated, and it is not possible to perform a 3D reconstruction of the patella by stereoradiography^(29)^. In addition, it is not feasible in all cases, because the patient must remain in the supine position for 10 s (for a lower-limb acquisition), a time similar to that required for the acquisition of a weight-bearing X-ray^(27)^.

## 3D BIPLANAR STEREORADIOGRAPHY AND THE CHALLENGES IN ASSESSING DEFORMITIES OF THE LOWER LIMBS

**Figure 2:**
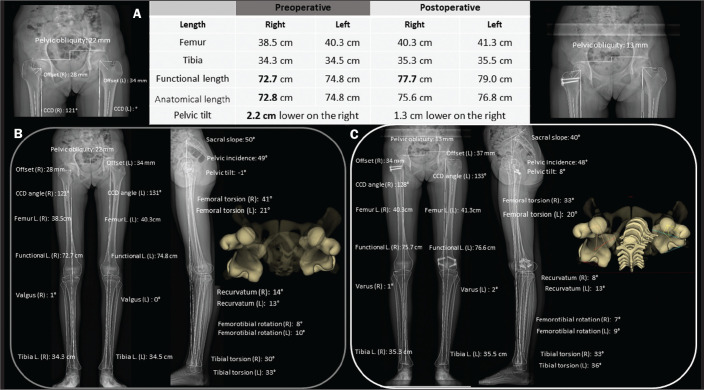
A: Preoperative and postoperative parameters in a 14-year-old female patient with pain and latent deformity of the right hip, with flattening of the femoral head, shortening of the femoral neck, and approximation of the greater trochanter in relation to the anteroinferior iliac spine. Preoperative evaluation by stereoradiography (B) showing recurvatum alignment (14° in the right knee and 13° in the left), a difference between the anatomical and functional lengths, and a 2.2 cm lower pelvic tilt on the right, together with accentuation of the right femoral torsion (anteversion of 41°). There was no angular deviation (valgus or varus). C: Postoperative epiphysiodesis showing a reduction in the difference in limb length and pelvic tilt (which was reduced to 1.3 cm). Reduction of the right recurvatum alignment (from 14° to 8°), as well as of the right femoral anteversion (from 41° to 33°, 3D axial view from above) was demonstrated. CCD, caput-collum-diaphyseal; L., length.

Decisions regarding bone-lengthening procedures or soft tissue surgery to correct bone deformities, as well as regarding the therapeutic follow-up of such deformities, demand the precise characterization of the shortening and the angular deformities^(30)^. As depicted in Figure 2, stereoradiography has proven to be a useful tool for the preoperative assessment of the lower limbs^(17)^. Limb deformities often occur in more than one of the three planes, which represents a challenge in the 2D assessment. However, for didactic purposes, they are listed here one by one.

### Length discrepancies

**Figure 3:**
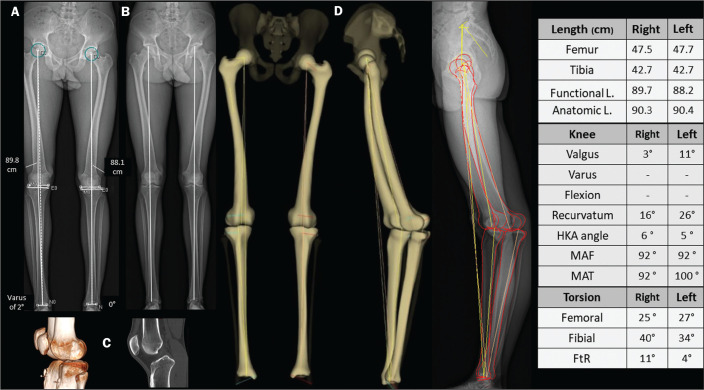
An evaluation performed exclusively in the frontal plane may be limited in cases of complex deformities, producing false results for the mechanical axes. Patient with bilateral recurvatum alignment on physical examination. A,B: A routine analysis only in the frontal plane showed a difference of 1.7 cm in the functional length of the lower limbs, 2° of mechanical varus malalignment of the right knee, and neutral alignment of the left knee. C: CT showing prominence of the anterior tibial tuberosity, with slight tendon ossification and hypoplasia of the tibial condyle on the left. In the 3D evaluation (D), it was evident that there is no difference in the anatomical length of the lower limbs (table). The sterEOS software determines the 3D frontal and sagittal planes for each knee, and the result was mechanical valgus of 3° on the right and valgus of 11° on the left, together with marked bilateral recurvatum alignment. L., length; HKA, hip-knee-ankle; MAF, mechanical axis of the femur; MAT, mechanical axis of the tibia; FtR, femorotibial rotation.

In the stereoradiographic assessment, the functional limb length is the direct 3D measurement of the distance between the center of the femoral head and the upper contour of the talar dome, therefore, including the knee articular interline, whereas the anatomical length is calculated by summing the 3D measurements of the femur (from the center of the head) and tibia, without taking into account the thickness of the articular interline. As a general rule, the functional lengths directly reflect the degree of pelvic tilt (measured between the acetabula). Therefore, in cases in which such measurements are discrepant, attention should be directed to the foot height in order to identify asymmetries in the plantar arches, which can also be measured by biplanar stereoradiography. On panoramic radiographs, misunderstandings regarding the limb length discrepancy may arise from their inability to measure differences in foot height^(31)^. Similarly, functional limb length less than the anatomical length should direct attention to deviations in valgus, varus, flexion, or recurvatum alignment^(31)^, as illustrated in Figure 3.

### Angular deviations

**Figure 4:**
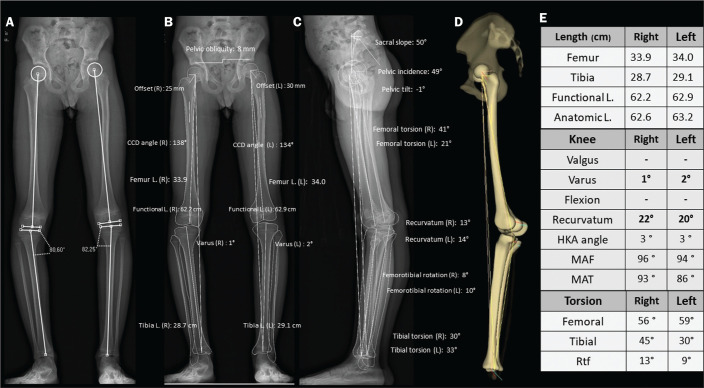
Seven-year-old patient, with joint hypermobility, under investigation for varus. Scanogram acquired in a standing position (A) showing bilateral tibial varus deformity (medial proximal tibial angle < 85°), with a femorotibial angle of 4° on the right and 3° on the left. It was not possible to evaluate recurvatum alignment. Low-dose biplanar stereoradiography (B,C) and corresponding 3D modeling (D) showing that there was no significant deviation of the mechanical axis (varus of 1° in the right knee and 2° in the left knee - E, Table) and demonstrating marked recurvatum alignment of both knees (22° on the right and 20° on the left), with increased femorotibial rotation, possibly secondary to joint hypermobility. The marked recurvatum alignment, together with the rotation, created the false impression of genu varus on the scanogram. L., length; HKA, hip-knee-ankle; MAF, mechanical axis of the femur; MAT, mechanical axis of the tibia; FtR, femorotibial rotation; CCD, caput-collum-diaphyseal; L., length.

The characterization of an angular deformity has two main requirements: the limb studied must be placed within a system of Cartesian planes in relation to a reference plane; the limb must be under a physiological load. In the lower limbs, the reference plane is the coronal plane of the knee, that is, the plane that is tangential to the posterior aspect of the femoral condyles and orthogonal to the ground. That defines how the knee is seen from the front and the degrees of deformity that should be understood as valgus/varus and flexion/recurvatum alignment. The medial proximal tibial and mechanical lateral distal femoral angles are automatically calculated from the 3D stereoradiography models. Because all measurements are based on 3D models of the limb, variations in positioning during acquisition do not affect the accuracy of stereoradiography. That gives stereoradiography a considerable advantage over panoramic radiography, which depends on the correct positioning, the reference being the patella centered on the femur, regardless of the position of the feet^(18)^. The success of this radiographic view depends on the availability of a fluoroscope for real-time positioning or on the experience of the radiology technician. Without those resources, the level of exposure to radiation is potentially increased. In addition, the patella is a reference that can be criticized, because, in cases of trochlear dysplasia or ligament laxity, it may be lateralized and will give an incorrect impression of the coronal plane of the knee (Figure 4), with consequent measurement errors^(32)^.

Studies have demonstrated the importance of evaluating axes under a load. In studies evaluating 20, 70, and 85 knees^(33-35)^, a two-degree difference between the femorotibial angle measured on images acquired in the standing position and that measured on those acquired in the supine position was found, as was a difference of 1.8° in the measurements of the joint line convergence angle, considered a predictor of the correction of coronal deviations after medial high tibial osteotomy, as well as an indicator of ligament laxity, making it clear that a study in the standing position is needed in order to complement the assessment of the degree of stability^(36)^.

**Figure 5:**
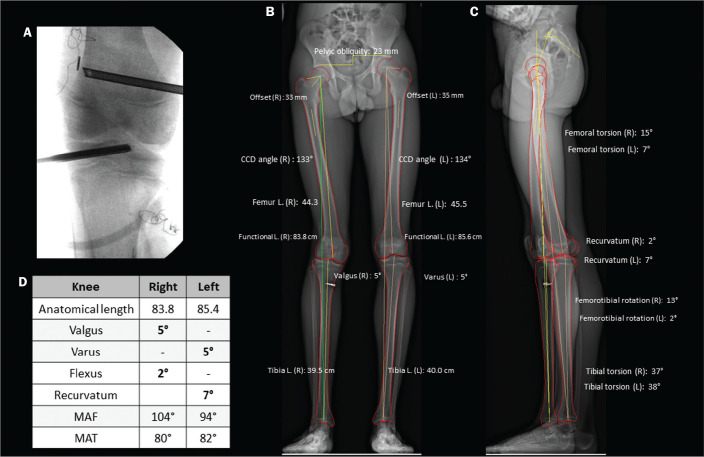
A 16-year-old male patient with gait alteration and with mild claudication on the right. A: Fluoroscopy for reconstruction of the anterior cruciate ligament of the right knee performed eight months prior. B,C: Stereoradiography showing valgus of 5° on the right and varus of 5° on the left, with femoral and tibial deformities, defined by abnormal mechanical lateral distal femoral and medial proximal tibial angles. There was a difference in the anatomical lengths of the left and right femurs (85.4 cm and 83.8 cm, respectively), albeit less pronounced than the pelvic tilt (explained by valgus), together with recurvatum alignment (D, Table). Progressive deformity of the right leg was associated with early closure of the lateral aspect of the distal femoral physis. In this case, stereoradiography facilitated the diagnosis and surgical planning, providing information beyond that obtained by conventional radiography. MAF, mechanical axis of the femur; MAT, mechanical axis of the tibia; CCD, caput-collum-diaphyseal; L., length.

In a recent study conducted by Jud et al.^(35)^, 2D projections were considered insufficient to represent the 3D anatomy of the joint. Therefore, substantial differences between 2D and 3D methods in the representation of the tibial plateau, as well as in the alignment of the lower limbs with or without weight-bearing, can lead to a considerably different impression of the preoperative anatomy (Figure 5), which can influence the surgical planning^(35)^. That study evaluated measurements of the femorotibial mechanical axis, comparing 2D biplanar stereoradiography of the entire limb with a scanogram in which the knees were extended in the supine position for computed tomography (2D without load) and with a 3D reconstruction of the same tomographic acquisition (3D without load). The authors concluded that considering both the 3D anatomical factors and those related to the standing position is a necessity in the planning of osteotomies^(35)^.

### Rotational deviations

Rotational and torsional abnormalities in the axial plane are poorly evaluated on conventional radiographic studies and are therefore underdiagnosed^(17)^. In addition to causes related to foot deformities (metatarsus adductus and valgus flatfoot), rotational abnormalities of the lower limbs may result from increased internal tibial torsion or femoral anteversion, as well as external tibial torsion or femoral retroversion^(37)^.

The measurements of femoral and tibial (bone) torsion provided by stereoradiography are equivalent to those of CT and are based on the same reference points described in the bimalleolar method defined by Reikerås et al.^(38)^, because that is the method that shows the greatest reproducibility. With that method, the evaluation of torsion can be carried out only in patients over five years of age, because it depends on the mineralization of the secondary ossification nuclei. From the 3D models, the two reference axes of the femur and the two reference axes of the tibia are automatically determined and projected in the axial plane to determine the degree of femoral and tibial torsion, respectively. The reference axes of the femur include a proximal axis, which passes through the center of the femoral head and the center of the base of the neck, and a distal axis, which is tangential to the posterior aspect of the femoral condyles. The reference axes of the tibia also include a proximal axis, which is tangential to the posterior aspect of the tibial condyles, and a distal axis, which is the line joining the center of the malleolus. Compared with CT, stereoradiography reduces the bias of selection of the axial slice to determine the axis of the femoral neck, referred to as the main difficulty and capable of strongly influencing the values of measurements on axial CT^(39)^.

To correlate the torsional deviations of the lower limb with the gait pattern, the index of cumulative torsions (ICT) is calculated^(40)^. The ICT is the sum of the femoral and tibial torsion angles, and by convention, the negative internal torsion (the femur commonly presents internal torsion) and positive external torsion (the tibia commonly presents external torsion). The ICT (normal range: +10° to +20°) reflects the degree of rotation of the foot during walking, being a dynamic angle (Fick angle, for which the normal range of external rotation is +5° to +18°). Adding a weak (negative) femoral torsion to a medium/high (positive) tibial torsion results in a high ICT (> +20°), whereas adding a medium/high femoral torsion to a weak tibial torsion results in a low ICT (< +10°). The static position of the foot in the anteroposterior radiographic view approximately reflects the step angle when the hips are neutral^(40)^.

When the ICT differs from the static position of the feet, it should be noted whether there are compensatory mechanisms in the knees or hips. More commonly, in cases with a low ICT (increased femoral torsion), there is internal rotation of the hip to reestablish the hip abductor moment arm, with consequences for the range of motion of the hip^(41,42)^. This pattern of compensation is not consistent across individuals, being more common in those with functional deficiency of the hip abductor moment arm, and may not manifest in patients with adequate gluteal muscle strength^(41)^. Within this chain of compensation, it is possible for the pelvis to adapt by anteversion (reducing the pelvic tilt angle and increasing the sacral tilt angle) or retroversion (as occurs in the biomechanical interdependence of the pelvis, hip, and spine), as described in various studies^(43)^. However, to our knowledge, there have been no studies directly assessing the effects that deformities of the lower limbs have on the position of the pelvis. In addition, other factors extrinsic to the femur and tibia should be considered, such as foot deformity (metatarsus adductus or metatarsus primus varus). These conditions can be additive or compensatory^(44)^.

A compensated torsional profile may lead to a false-negative perception of foot rotation on clinical assessment by simple physical examination or by 2D conventional radiography. Situations in which increases in femoral rotation (positive) are associated with marked tibial rotation (negative) may cause the axis of rotation of the foot to align with that of the direction of gait progression, reducing the clinical suspicion of rotational abnormalities. Painful manifestations related to friction syndromes and patellofemoral dysfunction may have their origins in rotational misalignments that are not visible on X-rays. When evaluating patients with patellofemoral pain, it is imperative to consider the rotational profiles of the femur and tibia^(45)^, which will inform the therapeutic decision-making process.

### Image evaluation proposal

**Figure 6:**
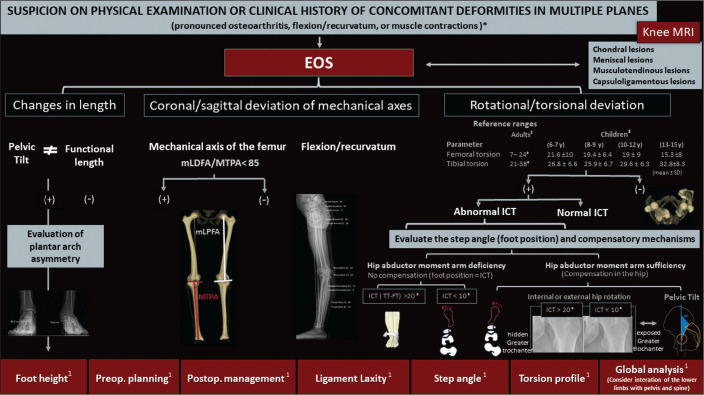
Proposal for the flow of patients with suspected deformity in more than one orthogonal plane. Biplanar stereoradiography can provide data about foot height, the torsional profile, and the step angle, with accurate and validated measurements. After image acquisition, radiologists should determine each set of measurements related to lengths (functional and anatomical), as well as their relationship with the pelvic tilt and foot height, together with deviation of the mechanical axis (in the coronal and sagittal planes) and the torsional profile (tibial torsion, femoral torsion, and femorotibial rotation), in order to characterize the deformity in its entirety. mLDFA, mechanical lateral distal femoral angle; MPTA, medial proximal tibial angle; mLPFA, mechanical lateral proximal femoral angle; TT, tibial torsion; FT, femoral torsion; Preop., preoperative; Postop., postoperative.

An overview of some of the data provided by the system is presented in Figure 6. The figure also shows a proposal for the flow of patients through the evaluation process.

* Examples of contexts in which to suspect concomitant deformities in multiple planes.

^1^ Data and applications that stereoradiography can provide.

^2^ Reference values for a torsional profile in adults^(18)^.

^3^ Reference values for a torsional profile in children^(18,27)^.

## FINAL CONSIDERATIONS

Various studies have shown that there is a high incidence of concomitant deviations of the lower limbs in more than one orthogonal plane. These combinations in varus and valgus with flexion or recurvatum alignment and rotational deformity reduce the accuracy of conventional radiography in measuring lengths and axes, because the method produces a 2D image based on the projection of a volume onto a reference plane, therefore being highly position-dependent, as well as having inherent magnification. Despite providing information in 3D, CT is limited by its high level of radiation and its inability to examine a patient in a functional (standing) position. Stereoradiography allows the characterization of multiplanar deformities under a load, with little dependence on patient position. Accurate measurements are the basis for more appropriate planning of the treatment of deformities of the lower limbs, with better results and lower complication rates. In addition, by enabling a broad study of the limbs or even the total body, it provides new perspectives on the recognition of compensatory mechanisms and the relationships among the spine, hips, and lower limbs.
